# An empirical study of the impact of environmental regulation on the eco-efficiency of digital agriculture: a quasi-natural experiment based on China’s carbon emissions trading pilot policy

**DOI:** 10.1186/s13021-026-00449-x

**Published:** 2026-05-14

**Authors:** Zhaoyang Lu, Diao Gou, Hailong Feng, Jianglai Dong, Nan Li

**Affiliations:** 1 School of Economics, Southwest University of Political Science and Law, Chongqing, 401120 China; 2 School of Business, Southwest University of Political Science and Law, Chongqing, 401120 China

**Keywords:** Environmental regulation, Digital agriculture, Eco-efficiency, Carbon emissions trading pilot policy, Eco-efficiency of digital agriculture, Dynamic DEA-Malmquist index, Q18 Q52 Q58 O13 C23

## Abstract

Carbon emissions trading systems have become increasingly prevalent amid rising global climate concerns and serve as key market-based tools for sustainable transformation. Agriculture is central to advancing China’s “dual carbon” strategy, requiring both emission control and reduction, while rapid digital agricultural development enables more precise carbon monitoring and management. This study examines whether China’s pilot carbon emissions trading pilot policy improves the eco-efficiency of digital agriculture. Using the dynamic data envelopment analysis (DEA)-Malmquist index method, we construct an evaluative framework to measure digital agricultural eco-efficiency, and based on panel data from 30 Chinese provinces over the period 2011–2022, we employ a difference-in-differences (DID) model to identify the policy effects. The empirical findings demonstrate that the ecological efficiency of China’s digital agriculture has successfully increased because of the implementation of the pilot policy for carbon emissions trading, and this conclusion passes several robustness tests. Heterogeneity analysis indicates that the effects of the policy vary across regions and different levels of development of digital agricultural eco-efficiency. According to the results of the mediating effect analysis, the pilot carbon emissions trading pilot policy increases forest coverage, which in turn increases digital agricultural eco-efficiency. The results of this research offer guidance for promoting environmentally sustainable agricultural development in China and for supporting international initiatives aimed at lowering agricultural greenhouse gas emissions.

## Introduction

Extreme weather events frequently occur because global climate change is intensifying, posing a serious threat to the stability of global ecosystems [1, 2]. This reality creates significant challenges for global food security and agricultural sustainability, exacerbating problems such as water shortages, soil erosion, and variability in crop production. The development of these issues extends across the entire agricultural value chain. At the same time, agriculture still generates large amounts of greenhouse gases [3], which makes it essential for achieving carbon neutrality worldwide. One of the largest agricultural countries is China, and it has long used agricultural methods that rely heavily on fertilizers and pesticides. Currently, it produces approximately 30% of the world’s carbon emissions [4]. As noted by Zhang et al. [5], agriculture is also closely linked to China’s entire economy; how well the agricultural sector works and how it grows are important for the country’s economic strength. This shows that there is a strong need for new technology and smart policies to support low-carbon agriculture and to move towards eco-friendly development [6, 7]. Some policies have helped agriculture in China become more eco-efficient, but China’s agriculture still falls behind that of richer countries in terms of how productive and advanced it is.

With the Industrial Revolution and globalization, the carbon emissions of many countries have increased [8]. Digital technology can accelerate the innovation process, facilitate the formation of consensus, and save time and costs; therefore, digitalization is gradually becoming an effective tool for reducing pollution [9, 10]. As digital tools become more common, many parts of agriculture now use big data, smart machines, and precise agricultural methods. These tools help farms produce more, solve environmental problems in agriculture, and make it easier to bring digital systems into important agricultural steps. The use of digital technology in agriculture helps address the natural limits of traditional agriculture and makes it more eco-friendly. To help the country move towards green development, the policy for trading carbon emissions is a crucial instrument [11, 12]. It gives companies reasons to cut carbon emissions by using market rules, and it supports greener economic and social development. Since this pilot policy started in 2013, it has helped reduce pollution, improve energy use, and promote more sustainable growth.

The academic community has formed two core research frameworks regarding the interaction between the carbon emissions trading pilot policy and agriculture: one focuses on “agricultural carbon reduction”, analysing the inhibitory effect of the carbon emissions trading pilot policy on total agricultural carbon emissions, the heterogeneity of emission reduction in different agricultural sub-sectors, the path through which the policy guides farmers to adopt low-carbon technologies, and the effectiveness of specific emission reduction mechanisms. The other focuses on “agricultural economic transformation”, paying attention to the impact of the policy on agricultural output value and labour productivity, the additional income that farmers obtain through participation in carbon sink transactions and low-carbon production, and the income differentiation effect of the policy on different-sized farmers. However, existing research focuses mostly on the one-way influence of the carbon emissions trading pilot policy on traditional agricultural carbon reduction, economic performance, or macro-level green transformation or limits the development effectiveness of digital agriculture to the single dimension of production efficiency improvement and technology diffusion. Few results incorporate the institutional incentives of carbon emissions trading policies and the improvement in the ecological efficiency of digital agriculture into a unified analytical framework, and systematic empirical tests on the causal relationship and internal mechanism between the two are lacking.

Addressing the research gaps above, this study argues that the three core areas of the micro-level logic, multi-dimensional measurement, and transmission path for enabling the high-quality development of digital agriculture through the carbon emissions trading pilot policy urgently need to be improved: The literature has not constructed an ecological efficiency evaluation system that is adapted to the “digital input–green output–environmental constraints” characteristics of digital agriculture, has failed to identify the policy effect of the carbon emissions trading pilot policy on the ecological efficiency of digital agriculture and the regional heterogeneity of this effect, and has ignored the “carbon trading policy–forest coverage improvement–optimization of digital agricultural ecological efficiency” key mediating transmission mechanism, making it difficult to reveal the deep coupling relationship between the carbon emissions trading pilot policy and digital agricultural ecological transformation. In response to these gaps, this study aims to answer the following questions:

1. Can the carbon emissions trading pilot policy affect the ecological efficiency of digital agriculture? Is the impact positive or negative?
2. If the policy can affect the ecological efficiency of digital agriculture, how heterogeneous is the impact across regions?
3. If the policy can affect the ecological efficiency of digital agriculture, what is the underlying mechanism?

In this paper, an integrated analysis framework for the carbon emissions trading pilot policy and the ecological efficiency of digital agriculture is theoretically constructed, the mediating mechanism of forest coverage is identified, and the measurement system for ecological efficiency adapted to the characteristics of digital agriculture is improved. Based on provincial panel data, we use a difference-in-differences (DID) model and a mediating effect model to identify the net effect and mediating effect of the policy, compensating for the lack of causal tests on the transmission path and providing a decision-making basis for optimizing carbon emissions trading system design and formulating differentiated green development strategies for digital agriculture in practice.

The structure of this paper is as follows. The introduction, included in Sect. “Introduction”, provides background information and the motivation of this study and presents the research questions. In Sect.  “Literature Review and Conceptual Framework”, the literature on agricultural eco-efficiency and environmental regulation is reviewed, important research gaps are noted, and a conceptual framework with testable hypotheses is developed. The study methodology is described in Sect.  “Methodology and Data”, which also covers data sources, empirical methods based on the DID and synthetic difference-in-differences (SDID) approaches, and the evaluation of digital agricultural eco-efficiency. A discussion of the policy implications follows the presentation of the empirical data in Sect.  “Empirical Results”, including baseline estimations, robustness checks, heterogeneity, and mechanism analyses. Section  “Discussion” is dedicated to an in-depth discussion, which interprets the key findings in relation to the literature. A summary of the main conclusions of this study, theoretical advancements, and recommendations for further research is provided in Sect.  “Conclusion and Policy Implications”.

## Literature review and conceptual framework

### Environmental regulation and agricultural eco-efficiency

Scholars and officials have taken a keen interest in China’s carbon emissions trading pilot policy. According to Jiang et al. [13] and Fan and Todorova [14], existing research has focused mostly on the main issues related to carbon pricing strategies, market-based instruments, and overall policy. The central purpose of the pilot policy is, through the use of market-based mechanisms, to hold industries accountable for reducing carbon emissions, but there is insufficient engagement in examining the long-term sustainability and stability of emission reduction [15]. Recent research has also emphasized how environmental regulation during complex external shocks, such as the COVID-19 pandemic, can still significantly reduce emissions under stringent enforcement mechanisms [16], highlighting the robustness of well-designed environmental policies.

Carbon pricing is an important component of the ETS since it signals the functioning of the market. Song et al. [17] and Wu et al. [18] have examined how market fluctuations are based on supply and demand, government expectations, and the business costs of emission reduction, which serve as drivers of fluctuating carbon prices. With respect to policy implications, carbon trading has a positive effect on reducing emissions and transforming industries towards green technologies [19, 20]. Furthermore, Chang et al. [21] conducted an additional analysis of the dynamic linkage effect of energy prices and carbon quota prices in regional carbon emissions trading pilot projects. They concluded that the two had a significant dynamic correlation and that the price linkage was significantly influenced by the market mechanism. Corporations are more inclined to reduce their carbon intensity and pollution in pilot regions when the quota allocation and incentive structures are well calibrated [22–24]. Furthermore, some researchers discovered that this was the case with the low-carbon city pilot programme, which lowered urban carbon emissions and encouraged the growth of green technologies [25, 26]. In 2022, Liu et al. used a DID model and confirmed that such policies have significantly advanced emission reduction at the municipal level [27].

The growth of non-fossil sources of energy and clean energy is another positive and significant success emerging from China’s carbon emissions trading scheme [28, 29]. Not only are these outcomes noticeable in the industrial sector with short-term emission reductions, but economic advantages also arise through the reduced or optimized consumption of these resources. The carbon emissions trading pilot policy has enabled businesses to implement low-carbon technology, economize resource use, and prevent high emissions [30–32]. Recent studies suggest that carbon trading positively influences green innovation through green patent applications [33–35]. The opportunity for change has possibly solidified China’s future in the global green economy [36], with additional economic revenue possibly arising from the policy. Moreover, debates at the international level caution against overestimating the potential of carbon markets, particularly in land use sectors, noting that technical limits and certification frameworks may restrict the long-term credibility of such mechanisms [37].

The implications of this research are encouraging for carbon emissions trading outcomes, but we need to consider how the findings of this research are related to the Porter hypothesis, which holds that environmental laws foster competition and innovation [38]. Evidence from the pilot policy in China supports this theory and suggests that regulatory policies aimed at reducing emissions will incorporate technological innovation. Compared with other carbon emissions trading sectors, certain industrial sectors have demonstrated much more significant reductions, indicating a differential capability and effectiveness within the overall carbon emissions trading pilot policy [39]. The contrasting outcomes indicate that policies need to consider regional and industrial differences to generate a functional carbon emissions trading system. Luan et al. [40] suggest that the policy will have an impact on overall TFP, indicating that the production processes or resource management of firms may be improved by the uptake of the carbon emissions trading pilot policy. Overall, the economic impacts seem to be large in regions with more fully functioning carbon markets, meaning that we will see both economic and environmental outcomes. The evidence ties back to the idea that market-based environmental governance may play more of a role in developing sustainable economic growth to engage with climate change in a global context [41, 42]. Supporting this idea, Fatima et al. [43] emphasize that environmental policy stringency is a critical factor in enabling an effective energy transition, suggesting that countries with stronger regulatory frameworks are more likely to realize sustainable outcomes.

While the emissions trading framework effectively lowers compliance costs and allows for more efficient emission reduction pathways [44], the successful application of carbon emissions trading relies on overcoming several barriers. High transaction costs, technical issues, and policy uncertainty limit the engagement of larger corporations [45]. These problems indicate that the carbon emissions trading arrangement will necessitate ongoing review and modification as a result of ensuring that systems can accommodate new technologies and, inevitably, social and economic growth. In addition, for carbon emissions trading systems to be sustainable in the future, regulations will need to change to reflect market developments, ensuring that policies remain relevant to the market [46].

Several studies on the impact of the policy have been conducted, especially in regard to emission reduction, company performance, and the operation of market mechanisms. A summary of the literature on China’s carbon emissions trading pilot policy is detailed in Table 1. Although existing studies provide extensive evidence on carbon pricing, green innovation, and emission reduction outcomes, several important gaps remain. First, most research concentrates on industrial sectors, aggregate agricultural emissions, or macro-level carbon intensity, with limited attention to digital agriculture as an emerging production mode that integrates data-driven inputs with ecological outputs. Second, the literature primarily evaluates emission reduction levels, while multi-dimensional efficiency indicators, especially eco-efficiency that incorporates digital technology and environmental performance, are rarely examined. Third, despite the recognition of regional heterogeneity in policy effectiveness, the specific transmission channels through ecosystem factors, such as forest coverage or land use dynamics, remain underexplored.

**Fig. 1:**
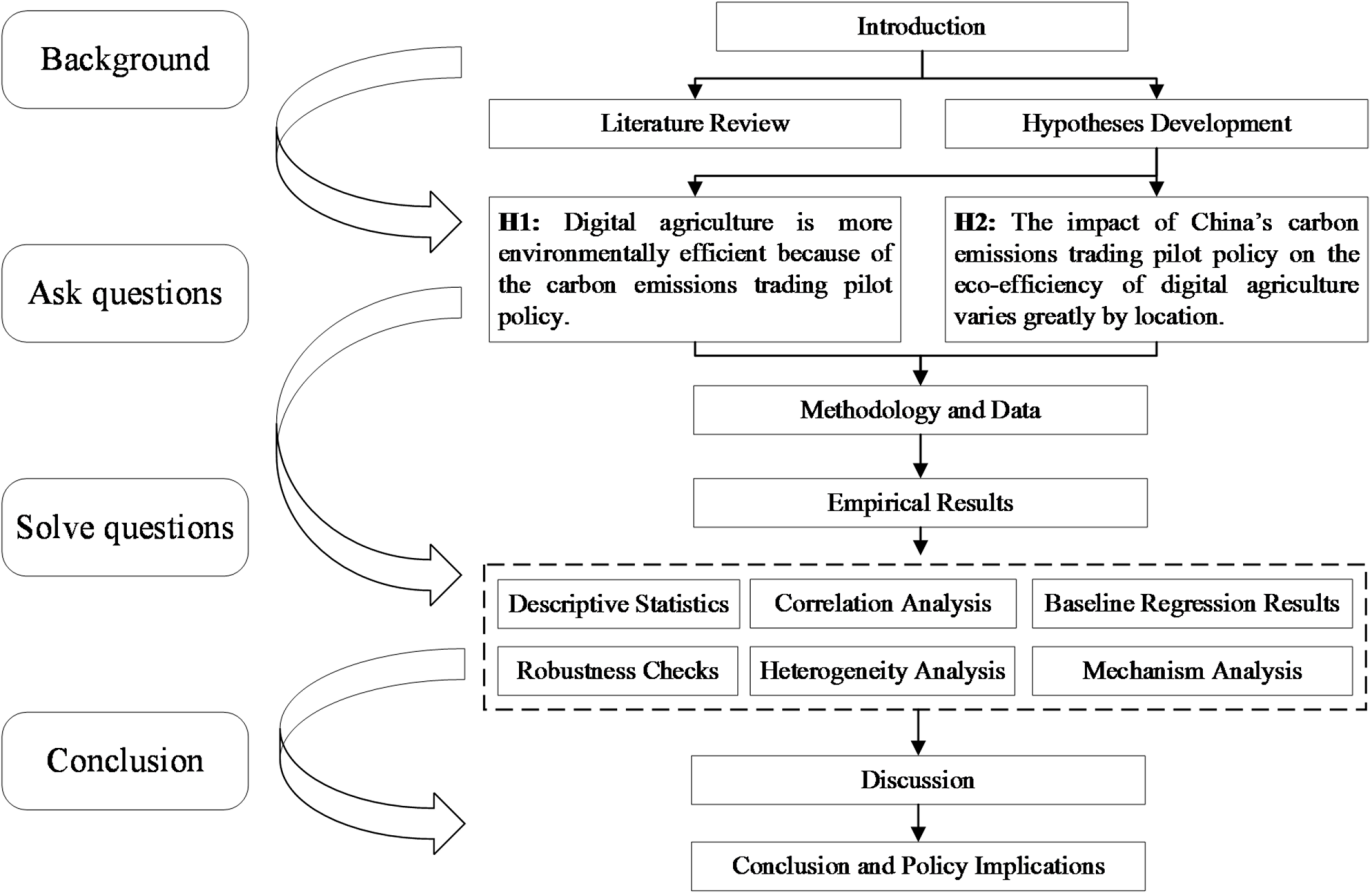
Conceptual Framework

This study leverages macro-level provincial data to empirically assess how the carbon emissions trading pilot policy affects digital agricultural eco-efficiency, providing empirical evidence to inform policymakers when they design targeted measures for green agricultural transformation. As a further contribution, this study integrates digital agriculture into eco-efficiency analysis. Few studies have combined these two areas to systematically evaluate digital agricultural eco-efficiency. Panel data from 30 Chinese provinces between 2011 and 2022 are used in this study to evaluate and analyse the eco-efficiency of digital agriculture by employing the dynamic data envelopment analysis (DEA)-Malmquist index method, which fills an existing research gap. A diagram of the framework of this study is displayed in Fig. 1. Beginning with the research background, the literature review motivates two testable hypotheses on the causal impact of China’s carbon emissions trading pilot policy on digital agricultural eco-efficiency and the regional heterogeneity of this impact. These hypotheses guide the empirical design, including model specification and data selection; baseline estimation, robustness checks, heterogeneity tests, and mechanism analysis are then conducted. The framework clarifies the identification logic linking the policy intervention to empirical inference and policy implications.

**Table 1:** Summary of the literature review

Research Theme	Research Focus	Key Findings	Representative References	Research Gap and the Contribution of the Studies
Policy Design and Effectiveness	Design logic, implementation outcomes, and stability under external shocks	China’s carbon ETS promotes emission reduction via market-based mechanisms. Even under crises such as the COVID-19 pandemic, strict enforcement yields positive effects.	[13, 14, 15, 16, 20, 22, 25, 26, 27, 43]	Extends ETS research beyond industrial sectors by examining digital agricultural eco-efficiency using provincial panel data with DID and dynamic DEA-Malmquist methods.
Market Mechanism and Carbon	Supply and demand dynamic pricing, price fluctuation, quota allocation, and incentive effects	Carbon pricing reflects market efficiency. Price volatility is driven by demand, policy expectations, and abatement costs. Proper quotas and incentives help firms reduce emissions.	[17, 18, 19, 21, 23, 24, 44]	Moves beyond carbon price analyses to evaluate the ETS from a productivity–eco-efficiency perspective, measuring efficiency changes in agriculture.
Green Innovation and Productivity	Influence of the ETS on green technology adoption, patents, and TFP	The ETS drives technological upgrading and green innovation, especially in regions with active markets. Green patents rise; TFP improves. Supports the Porter hypothesis of innovation under regulation.	[30, 31, 32, 33, 35, 40, 41, 42]	Links the ETS to digital agricultural transformation and eco-efficiency, offering new evidence for the Porter hypothesis in the agricultural context.
Economic and Environmental Synergy	Co-benefits: energy transition, resource use, economic gains	The ETS leads to optimized energy consumption and better resource efficiency and enhances firms’ economic performance while reducing emissions. Strong markets yield greater dual benefits.	[29, 34, 38, 39, 43]	Identifies regional and development-stage heterogeneity in ETS effects on digital agriculture, revealing spatial disparities in policy outcomes.
Limitations and Policy Refinement	Barriers to the ETS: transaction costs, uncertainty, technical constraints	High costs, unclear rules, and technical limitations hinder participation. Continuous policy updates and adaptive design are essential. Carbon markets in land use face certification challenges.	[37, 45, 46]	Uncovers an ecological mechanism whereby the ETS improves eco-efficiency via increased forest coverage, addressing underexplored transmission channels in agricultural ETS studies.

### Hypothesis development

Many nations have set goals to reduce carbon emissions because of the escalation of climate change worldwide. Agriculture generates a large amount of carbon and has a significant opportunity to lower these emissions [47–49]. The carbon emissions trading pilot policy incorporates financial incentives to encourage companies to reduce emissions. Although China emits the most carbon worldwide, it has tried out and used the carbon emissions trading pilot policy to achieve eco-efficiency. Under the framework of this policy, these initiatives not only offer innovative solutions to climate change but also create new avenues for promoting green transformation and cutting carbon emissions. Put differently, the policy encourages agricultural enterprises to invest in more sustainable practices and technology while lowering their carbon emissions, which can help them earn additional income through the sale of carbon credits and contribute to the achievement of environmental goals [50].

The carbon emissions trading pilot policy becomes more important as digital agriculture grows fast. Digital agriculture involves the application of innovative tools to enhance the eco-friendliness of agricultural practices; these tools include precision planting, intelligent irrigation, and drones. By optimizing resource utilization and reducing carbon emissions, they contribute to a more sustainable agricultural sector. The carbon emissions trading pilot policy sets clear goals for reducing emissions, encourages agricultural businesses to adopt digital tools, and helps them optimize their operations and resource use. This, in turn, creates a more efficient and environmentally friendly approach to farming [51].

Additionally, another significant factor in this change is governmental support. The government provides financial subsidies, technical training, and information services to help agricultural businesses use new technologies, which helps encourage agriculture to use more digital tools. This change not only gives agricultural enterprises more chances to develop but also helps them perform better in the ETS. The ETS limits future carbon emissions and makes enterprises more willing to cut their emissions. In this market, agricultural companies deal with higher costs, and they also need to meet new regulations to lower their emissions. Because of both regulatory requirements and market competition, agricultural businesses are working harder to cut emissions, which helps agriculture move towards low-carbon growth [52, 53]. Based on these ideas and facts, the following study hypothesis is proposed:

#### Hypothesis 1

**(H1)**: Digital agriculture is more environmentally efficient because of the carbon emissions trading pilot policy.

A market-based approach to lowering greenhouse gas emissions is the carbon emissions trading pilot policy, which offers financial incentives through market mechanisms. However, as Halperin et al. [54] and Liu et al. [55] note, we still do not fully understand how this policy works in different places or for types of agriculture. In agriculture, the effects of the policy are not the same everywhere; there are significant differences between regions, especially in terms of how well digital agriculture works. Digital tools are seen as very helpful for clean agriculture, aiding in resource allocation, reducing emissions, and increasing productivity [56]. However, to use these tools well, good technologies and strong support systems are essential. In richer areas with advanced technology and digital systems, the policy works better. These places can use digital tools more, which helps agriculture become more eco-friendly. However, in poorer regions, weaker systems and the lower rate of adoption of technology can diminish policy effectiveness. This leads to a significant reduction in the overall benefits that the policy can achieve, a phenomenon known as the “policy fatigue effect” [57].

Furthermore, in richer places with more advanced technology, the agricultural sector uses tools such as smart watering systems, high-tech machines, and drones, which help farms develop more and pollute less. In contrast, in poorer areas, there are not enough tools, systems, or support to use digital agriculture in the same way, which makes the policy less helpful for eco-friendly agriculture. Moreover, regions with greater access to advanced technologies and research are better able to deal with changes. These areas also tend to develop new, environmentally friendly agricultural technologies more rapidly, which enables them to transition to digital agriculture more swiftly and achieve greater reductions in carbon emissions. However, places without this face slow technology growth, making the policy less useful and slowing progress towards clean agriculture. Because of these differences across regions, the way in which the carbon emissions trading policy works also varies from place to place. In light of the analysis above, Hypothesis 2 (H2) is proposed:

#### Hypothesis 2

**(H2)**: The impact of China’s carbon emissions trading pilot policy on the eco-efficiency of digital agriculture varies greatly by location.

## Methodology and data

### Measurement of digital agricultural eco-efficiency

In this study, input–output indicators are established by adapting the agricultural eco-efficiency evaluation framework introduced by Yu et al. and Yang et al. [58, 59]. To enhance the evaluation, additional first-tier indicators reflecting the digital agricultural environment have been integrated. Key indicators include the number of businesses functioning within the software and IT services industry, the aggregate telecommunications business volume, and the total length of fibre-optic cable infrastructure. This approach aims to develop China’s digital agricultural eco-development indicator system [60].

The proposed indicator system is structured around three first-tier input indicators: the digital agricultural environment, digital agricultural resource inputs, and digital agricultural eco-development. It further incorporates a single first-tier output indicator: digital agricultural benefits. The digital agricultural environment metric is designed to evaluate the essential prerequisites for advancing a digitally driven agricultural ecosystem. The growth of digital infrastructure and the degree of information technology integration in agricultural production processes are its two primary points of focus. Digital agricultural resource inputs represent the factors driving digital agricultural eco-development, including key agricultural inputs such as mechanization, arable land, and water resources. Digital agricultural eco-development represents the sustainability of agricultural development and includes factors representing the core of high-quality digital agricultural progress. Digital agricultural benefits are the production goal of digital agricultural eco-development and measure the returns from digital resource investment and agricultural resource utilization. Table 2 presents this study’s input–output indicator system for China’s digital agricultural eco-development.

**Table 2:** Input–output indicator system for China’s digital agricultural eco-development

Indicator	Level One Indicator	Level Two Indicator	Quality
Inputs	Digital Agricultural Environment	The number of software and information technology services enterprises	+
		The total volume of telecommunication services	+
		Fibre-optic cable line length	+
		The number of businesses engaged in e-commerce trading	+
		Postal outlets	+
		E-commerce purchases	+
	Digital Agricultural Resource Inputs	Total agricultural machinery power	+
		Total area of crops planted	+
		Effective irrigated area	+
	Digital Agricultural Eco-Development	The quantity of fertilizer applied to crops	-
		Pesticide use	-
		The quantity of plastic film utilized in agriculture	-
Expected output	Digital Agricultural Benefits	Retail sales of agricultural products online	+
		E-commerce sales	+
		The total value of production from agriculture, forestry, fishery, and animal husbandry	+

Different indicators have different units and properties, requiring standardization to eliminate dimensional effects and to ensure comparability across variables. Standardization is conducted via different formulas for positive and negative indicators, as shown in Eqs. (1) and (2), respectively:

1 $$\begin{aligned}\rm{Positive\; indicators:} {X_{ij}}\\=\frac{{{x_{ij}} - \hbox{min} ({x_{ij}})}}{{\hbox{max} ({x_{ij}}) - \hbox{min} ({x_{ij}})}}\end{aligned}$$

2 $$\begin{aligned}\rm{Negative\; indicator:} {X_{ij}}\\=\frac{{\hbox{max} ({x_{ij}}) - {x_{ij}}}}{{\hbox{max} ({x_{ij}}) - \hbox{min} ({x_{ij}})}}\end{aligned}$$

The Jth index value of the province and the value following standardization are represented by the two formulas. A larger positive indicator is considered better; a smaller negative indicator is also considered better in this way.

Farrell laid the foundation for DEA in 1957, and Charnes et al. subsequently formalized it as a non-parametric efficiency assessment method in 1978 [61, 62]. DEA employs convex analysis and mathematical programming to calculate the relative efficiency of decision-making units (DMUs), especially when several inputs and outputs exist. DEA makes it possible to evaluate performance without requiring specific functional forms or distributional assumptions, and it is inherently data driven, scale invariant, and unit free. Over time, the methodology has evolved significantly. The original model based on CRS has been expanded to VRS. Furthermore, the DEA has moved forward from using only fixed, cross-sectional models to using dynamic models with panel data. It now includes both desired and undesired results, which has led to a wide range of model variants and broader ways to employ them.

Malmquist was the first to propose the concept of a productivity index in 1953, and it was later codified by Caves et al. [63, 64]. Then, in 1992, Färe et al. developed the DEA-Malmquist index, representing important progress with regard to DEA [65]. This index combines DEA with ways to measure productivity by using input-based or output-based distance functions, showing how TFP changes over time. Moreover, the DEA-Malmquist index is frequently used to compare productivity between two time periods, usually from time t to t + 1 [66], as shown in Eq. (3).

3 $$\begin{aligned}M({x^{t+1}},{y^{t+1}},{x^t},{y^t})\\={[\frac{{{D^t}({x^{t+1}},{y^{t+1}})}}{{{D^t}({x^t},{y^t})}} \times \frac{{{D^{t+1}}({x^{t+1}},{y^{t+1}})}}{{{D^{t+1}}({x^t},{y^t})}}]^{\frac{1}{2}}}\end{aligned}$$

In the framework of the DEA-Malmquist index, $({x^t},{y^t})$ and $({x^{t+1}},{y^{t+1}})$ represent the input–output variable combinations for periods t and t + 1, respectively. The terms ${D^t}$ and ${D^{t+1}}$ denote the corresponding output distance functions; they represent the output variable’s maximal growth under a specified input variable within a given period [67]. Assuming CRS, two main components may be classified in the DEA-Malmquist index: EFFCH and TECHCH [68]. When the assumption of VRS is introduced, two separate components may be further separated from the EFFCH index: PECH and SECH. As a result, the following is the final breakdown of the DEA-Malmquist index under VRS, as shown in Eqs. (4) and (5):

4 $$\begin{aligned} M({x^{t+1}},{y^{t+1}},{x^t},{y^t})&=\frac{{{D^{t+1}}({x^{t+1}},{y^{t+1}})}}{{{D^t}({x^t},{y^t})}}\\ &\quad \times {[\frac{{{D^t}({x^{t+1}},{y^{t+1}})}}{{{D^{t+1}}({x^{t+1}},{y^{t+1}})}}{\times} \frac{{{D^t}({x^t},{y^t})}}{{{D^{t+1}}({x^t},{y^t})}}]^{\frac{1}{2}}} \end{aligned}$$

5 $$\begin{aligned}=Effch \times Techch\\Tfpch=Effch \times Techch\\=(Pech \times Sech) \times Techch\end{aligned}$$

When Techch > 1, productivity has grown due to technological advancements during this time. In contrast, when Techch < 1, it indicates that the technological progress during this period has prevented further improvement in productivity. When Techch = 1, technological progress between periods contributes little to productivity growth. In addition, technical efficiency is effective, and productivity is further enhanced when Effch > 1. Technical efficiency is inadequate when Effch < 1. Productivity is unaffected by technical efficiency when Effch = 1.

In addition, the dynamic DEA-Malmquist index is employed to better capture the evolving nature of digital agriculture under the carbon emissions trading pilot policy. Digital agricultural eco-efficiency is inherently path dependent, reflecting continuous adjustments in digital infrastructure, resource allocation, and environmental performance over time. Compared with static DEA models, the dynamic DEA-Malmquist framework allows an intertemporal linkage of inputs and outputs, measures changes in productivity across periods, and decomposes efficiency into technological progress and efficiency change components. This approach is particularly suitable for identifying the policy-induced productivity dynamics and structural adjustments associated with China’s carbon emissions trading pilot policy.

### Econometric strategy: a quasi-natural experiment approach

DID model. The DID method, which is a widely adopted econometric approach, effectively mitigates endogeneity concerns, thereby strengthening the reliability of causal inferences. In this research, the implementation of the carbon emissions trading pilot policy is seen as a quasi-natural experiment. The treatment group comprises six designated pilot regions; however, Shenzhen—a city-level administrative entity within Guangdong Province—is excluded to maintain consistency on a geographical scale at the provincial level. Because insufficient data are available, Tibet, Hong Kong, Macao, and Taiwan are not included in the control group, which consists of 24 non-pilot provinces. We employed a single-period DID model, as shown in Eq. (6), to assess the disparate changes in the eco-efficiency of digital agriculture between areas before and during the adoption of the policy. The goal of this empirical approach is to measure how the policy affects the development of the eco-efficiency of digital agriculture.

6 $$\begin{aligned} DATF{P_{it}}&=\alpha +\beta CET{R_{it}}\\ &\quad+\gamma Contro{l_{it}}\\ &\quad+{\mu _i}+{\lambda _t}+{\varepsilon _{it}}\end{aligned}$$

where $CET{R_{it}}$ indicates whether province *i* in year *t* has implemented the carbon emissions trading pilot policy and $DATF{P_{it}}$ denotes the digital agricultural ecological efficiency of province *i* in year *t*. The coefficient *β * is the main indicator used to assess the overall treatment impact of the pilot policy. The control variables are denoted as $Contro{l_{it}}$. Provincial fixed effects and year fixed effects are denoted as ${\upmu _i}$ and ${\uplambda _t}$, respectively. These fixed effects govern the temporal variables of each province and capture geographical traits that remain constant over time. The term ${\upvarepsilon _{it}}$ follows an independence and identical distribution and is the random error term.

**Fig. 2:**
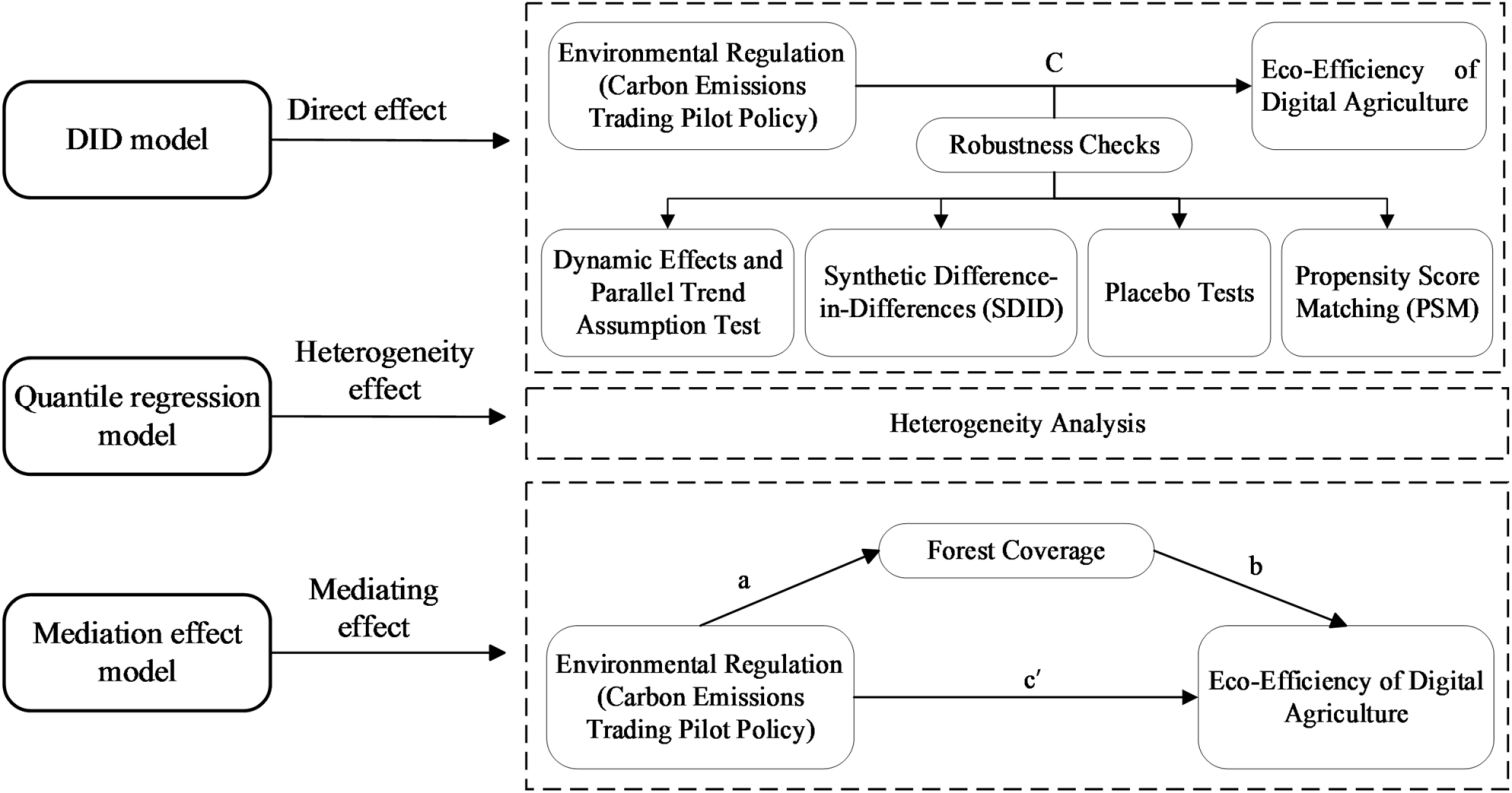
Analytical Strategy

Quantile regression model. In this paper, fixed effects panel quantile regression is employed. Beyond mitigating the impacts of outliers and capturing individual heterogeneity, it reveals heterogeneous policy effects across different digital agricultural eco-efficiency quantiles, addressing the limitation of mean regression, which overlooks asymmetric responses.

7 $$\begin{aligned}{Q_\uptau }(DATF{P_{it}}\,|\,Contro{l_{it}},{\mu _i},{\lambda _t})&=\eta \uptau +{\chi _\uptau }CET{R_{it}} \!\!\!\!\!\!\quad+{\varphi _\uptau }Contro{l_{it}}+{\mu _{i,\uptau }} \!\!\!\!\!\!\quad+{\lambda _{t,\uptau }}+{\varepsilon _{it,\uptau }}\end{aligned}$$

In Eq. (7), ${Q_\uptau }(DATF{P_{it}}|.)$ denotes the conditional distribution of digital agricultural eco-efficiency at the *τ * quantile; the remaining variables remain the same as above; and ${\chi _\uptau }$ is the policy impact coefficient of concern, which varies with the quantile. The regression situation at the corresponding quantiles can be obtained by setting different *τ * values, and the standard deviation of the parameters in the regression is obtained by the bootstrap method proposed by Buchinsky.

Mediating effect model. In this paper, in reference to Lu et al. [69, 70], a three-step test procedure for the mediating effect is used to analyse whether environmental regulation affects the eco-efficiency of digital agriculture by affecting the forest coverage rate.

8 $$\begin{aligned}FORES{T_{it}}&={\alpha _1}+{\beta _1}CET{R_{it}}\\ &\quad+{\gamma _1}Contro{l_{it}}\\ &\quad+{\mu _i}+{\lambda _t}+{\varepsilon _{it}}\end{aligned}$$

9 $$\begin{aligned}DATF{P_{it}}&={\alpha _2}+{\beta _2}CET{R_{it}}\\ &\quad+{\delta _2}FORES{T_{it}}\\ &\quad+{\gamma _2}Contro{l_{it}}\\ &\quad+{\mu _i}+{\lambda _t}+{\varepsilon _{it}}\end{aligned}$$

The other factors are in line with those above, and $FORES{T_{it}}$ serves as the mediating variable (forest coverage). In line with Eq. (6) above, the regression coefficient *β * of *DATFP* represents the impact of environmental regulation on the eco-efficiency of digital agriculture, and Eqs. (8) and (9) are the procedures for conducting the test of the mediating effect. The analysis strategy of this paper is shown in Fig. 2.

### Variable definitions and data sources

Dependent Variable: Digital Agricultural Eco-Efficiency (DATFP). Employing data from 2011 to 2022, this study assesses the digital agricultural eco-efficiency of 30 Chinese provinces via the dynamic DEA-Malmquist index method.

Independent Variable: Carbon Emissions Trading Pilot Policy (CETR). China’s NDRC announced the carbon emissions trading pilot policy for the first time in 2011. Subsequently, operational trading platforms were launched between 2013 and 2014. Following the approach adopted in prior research, the policy is assumed to have taken effect in the pilot regions beginning in 2013 [71]. To empirically capture the implementation of the policy, the CETR variable is constructed via the DID method by interacting with a geographic dummy variable and a temporal dummy variable. Specifically, if a province has been classified as a carbon trading pilot region, the regional dummy takes a value of 1; otherwise, it takes a value of 0. Similarly, the time dummy is coded as 1 for 2013 and onward and 0 for the pre-policy period.

Mediating Variable: Forest Coverage (FOREST). The carbon emissions trading pilot policy can motivate local governments and relevant stakeholders to participate in afforestation and reforestation projects, thus improving forest cover, especially in rural and agricultural areas [72]. Research has shown that the carbon ecological carrying capacity of areas has greatly increased since the implementation of the carbon emissions trading pilot policy, and the forest coverage of regions with high carbon ecological carrying capacity is higher [73]. In addition, enhanced forest cover can lessen soil erosion, promote water retention, and lessen the adverse effects of extreme weather events on agriculture, thereby enhancing agricultural sustainability. As an ecological buffer zone, forests can reduce non-point source pollution, promote a cleaner and more stable agricultural environment, and further promote an improvement in ecological efficiency [74]. Following the literature [75], we used forest area/land area as a measure of forest coverage.

Control Variables: in accordance with previous studies [76, 77], we examine a range of control variables at the provincial level: the level of informatization (IL), the degree of openness (FO), research and development (R&D) intensity (RD), the industrial structure (IS), fiscal support for agriculture (AGR), the economic development level (ECO), environmental regulation (ER), population size (PS), and the educational development level (EDU). The control variables are selected based on production efficiency theory, environmental economics, and the literature on digital agriculture and carbon policy evaluation. IL, RD, and EDU capture technological progress and human capital, which improve eco-efficiency through innovation and factor substitution. ECO, FO, and IS reflect structural and market conditions that affect resource allocation and environmental performance. AGR and ER represent government intervention and regulatory pressure, which influence green investment and compliance behaviour, while PS controls for scale and resource demand effects. Table 3 lists the specific symbols and definitions of the main variables.

**Table 3:** Key variables and methods for calculation

Variable Category	Variable Name	Symbol	Definition
Dependent Variable	Digital Agricultural Eco-Efficiency	DATFP	The dynamic DEA-Malmquist index method is utilized to calculate the variable.
Independent Variable	Carbon Emissions Trading Pilot Policy	CETR	Policy dummy variable
Mediating variable	Forest coverage	FOREST	Forest area /land area
Control Variables	Informatization level	IL	IL=Total volume of posts and telecommunications services /GDP
	Degree of opening up	FO	FO= Gross import and export /GDP
	R&D intensity	RD	RD= Internal R&D expenditure /GDP
	Industrial structure	IS	IS = The proportion of secondary industry output value to tertiary industry output value.
	Fiscal support for agriculture	AGR	AGR= Total amount spent on water, forestry, and agriculture divided by the total fiscal budget
	Level of economic development	ECO	ECO = GDP per capita
	Environmental regulation	ER	ER = The logarithm of the province’s government work report’s overall text length about the severity of environmental regulations
	Population size	PS	PS = The population’s logarithm at the end of the year
	Educational development level	EDU	EDU= (Primary school ×6 + junior high school ×9 + senior high school ×12 + junior college and above ×16)/ population aged 6 years and above

Owing to data availability and the timeline of policy implementation, we chose a period that spans from 2011 to 2022, and the final panel dataset comprises 360 annual observations covering 30 Chinese provinces. The core data for calculating DATFP, which represents digital agricultural eco-efficiency, are sourced from the China Statistical Yearbook, China Rural Statistical Yearbook, China Tertiary Industry Statistical Yearbook, and the official statistical yearbooks of the respective provincial-level regions. Additional factors can be found in the CSMAR database. To ensure data completeness, missing values are estimated via linear interpolation. The descriptive statistical findings for the primary variables are shown in Table 4.

**Table 4:** Descriptive statistical results

VARIABLES	(1)	(2)	(3)	(4)	(5)	(6)
	*N*	Mean	Sd	min	max	VIF
DATFP	360	1.1121	0.4506	0.2710	3.8827	-
EFFCH	360	1.0005	0.0791	0.4335	1.7687	-
TECHCH	360	1.1085	0.4288	0.2824	3.8827	-
IL	360	0.0584	0.0467	0.0203	0.1791	1.17
FO	360	0.2617	0.2473	0.0440	0.9133	3.61
RD	360	2.0910	1.4188	0.4924	5.5874	1.54
IS	360	1.2936	0.4902	0.7550	2.8100	2.14
AGR	360	11.3388	3.1854	5.1594	17.1347	3.97
ER	360	9.7882	0.1560	9.4928	10.0865	1.09
PS	360	8.2149	0.7128	6.5250	9.2109	1.46
ECO	360	5.7336	2.5837	2.6627	12.0177	3.00
EDU	360	9.2839	0.7693	8.0358	11.1923	3.20

## Empirical results

### Baseline regression results

To avoid invalid estimates due to multicollinearity between the explanatory variables, this study applies a VIF test to assess the model. The VIF values are less than 4.0, as shown in Column (6) of Table 4. This finding indicates that the explanatory variables of this study are appropriate. Table 5 displays the estimation findings. The baseline regression findings with the addition of provincial fixed effects alone, provincial fixed effects plus time fixed effects, and the two-way fixed effects and control variables are shown in Columns (1), (2), and (3), respectively. Across all model specifications, the coefficients of the interaction term are robustly positive, indicating that digital agricultural eco-efficiency is positively impacted by the policy, which supports Hypothesis 1.

**Table 5:** Results of the baseline regression

VARIABLES	(1)	(2)	(3)
	DID_DATFP	DID_DATFP	DID_DATFP
CETR	0.3812***	0.2425**	0.2808**
	(0.0791)	(0.0895)	(0.1155)
Constant	1.0486***	0.9554***	-1.9140
	(0.0132)	(0.0355)	(3.7602)
Observations	360	360	360
Control variables	NO	NO	YES
Year FE	NO	YES	YES
Province FE	YES	YES	TES
R-squared	0.0210	0.6210	0.6310
ID Number	30	30	30

Standard errors are in parentheses. *** *p* < 0.01, ** *p* < 0.05, * *p* < 0.1

As a regulatory tool that is based on the market, the carbon emissions trading pilot policy can promote the employment of low-carbon management techniques in the agricultural sector and encourage investment in more efficient production technologies. By guiding resources towards organizations that prioritize low-carbon and efficient practices, the policy helps to create an environment that is conducive to the growth of sustainable agriculture. In terms of digital agriculture, this resource optimization may facilitate the deployment of advanced digital technologies, thereby contributing to improved ecological performance and promoting green agricultural transformation.

### Robustness checks

Several robustness tests are carried out to verify the empirical results. The results of these tests consistently reveal how China’s carbon emissions trading pilot policy significantly increases the eco-efficiency of digital agriculture.

#### Dynamic effects and the test of the parallel trends assumption

The DID model is predicated on the idea that the change trends of the treatment group and the control group were identical before the introduction of the policy. We use the parallel trends test to verify whether the parallel trends are accurate. The model is shown as Eq. (10):

10 $$\begin{aligned}DATF{P_{it}}&=\alpha +\sum\nolimits_{{ - 2}}^{{ - 1}} {{\beta _i}CETR\_Befor{e_{it}}}\\ &\quad +\sum\nolimits_{1}^{5} {{\beta _i}CETR\_Afte{r_{it}}} +\gamma Contro{l_{it}}\\ &\quad +{\mu _i}+{\lambda _t}+{\varepsilon _{it}}\end{aligned}$$

**Fig. 3:**
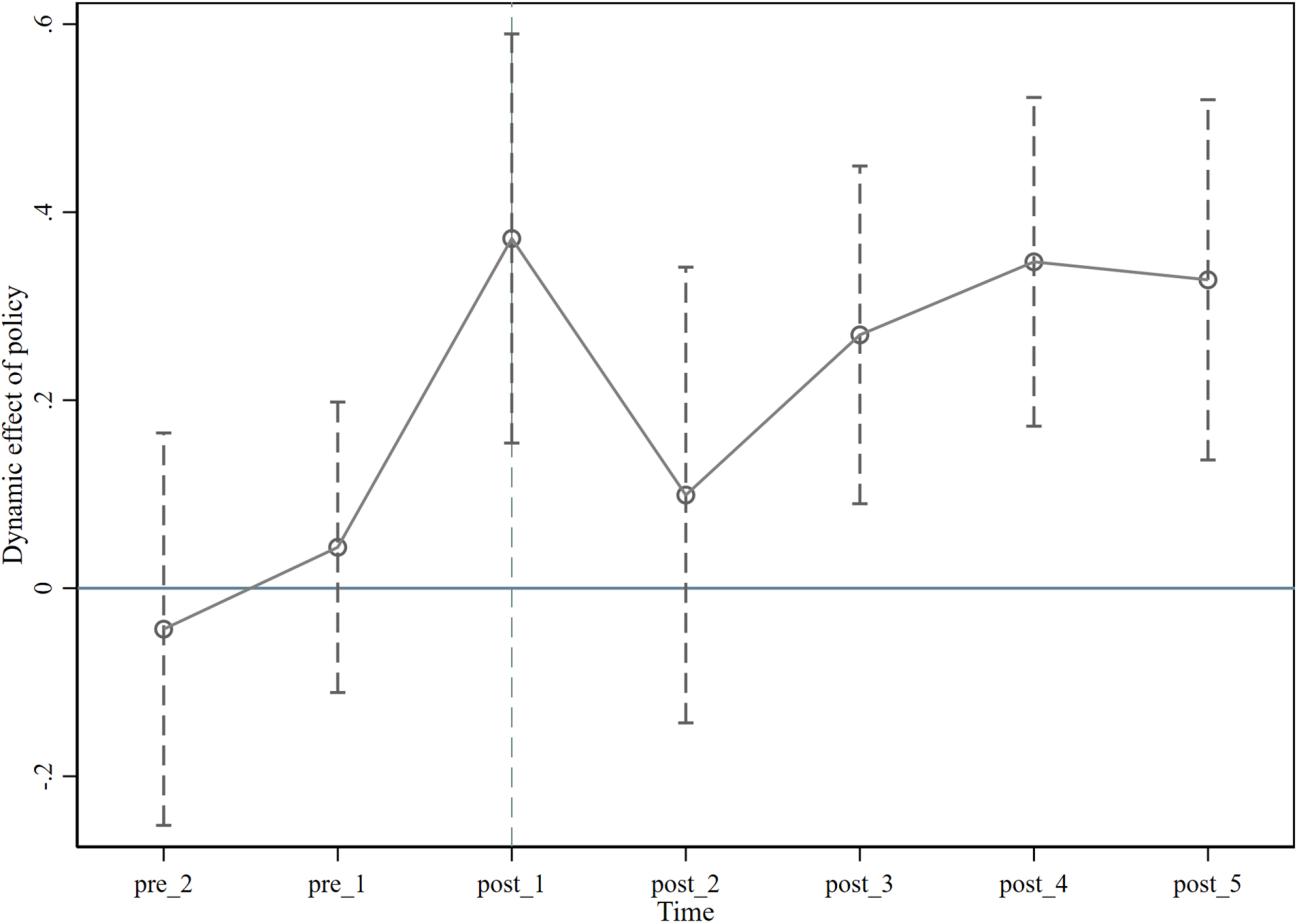
Parallel trends test and dynamic effects

Two years before and five years after implementation, as well as whether the area is on the carbon emissions trading list, the explanatory variables $CETR\_Before$ and $CETR\_After$ reflect the interaction terms of the dummy variables. Put differently, $CETR\_Afte{r_1}$ represents the first year after the introduction of the policy. The term $CETR\_Afte{r_5}$ represents five years after policy introduction. The other variables remain consistent with those above. In the parallel trends test, the baseline period is set to t − 1, which corresponds to the year before the introduction of the policy. We take 2013 as the base period and exclude it from this analysis, considering that the policy was formally announced at the end of 2013. If there is no significant difference between 0 and the coefficient *β * before the pilot, this result implies that both groups evolved similarly before the policy intervention. The calculated coefficients together with their 95% confidence intervals are presented in Fig. 3.

Before the implementation of the policy, there were no appreciable variations in the eco-efficiency of digital agriculture between the pilot and non-pilot regions, indicating that the parallel trends assumption is generally satisfied. Specifically, the estimated coefficients for 2011 and 2012 are not statistically different from zero. Although the coefficient in 2014 becomes significant, it is followed by a temporary return to insignificance in 2015, which may reflect short-term fluctuations or an adjustment period in the early stage of policy implementation rather than a systematic deviation from the parallel trend. Starting from 2016, the impact of the policy becomes consistently statistically significant, suggesting a delayed effect. This lag may be explained by the time required to establish effective policy incentives and a functioning carbon market. The formal launch of the policy at the end of 2013 provided initial price signals for carbon emissions, motivating enterprises and local governments to gradually adopt emission reduction strategies. In the initial phase, policy effects may have been partially absorbed by the market, accompanied by a period of adaptation and adjustment. As the pilot policy progressed, implementation details were refined, market mechanisms matured, and stakeholders better understood and responded to the policy, leading to more stable and pronounced effects over time.

#### Synthetic Difference-in-Differences (SDID)

The validity of the findings must be carefully examined, even if the test of the parallel trends assumption indicates a passing result, because this test does not guarantee that this similarity persists after the policy is implemented. Therefore, passing the test of the parallel trends assumption by itself might not be enough to confirm the DID results; the unverifiability in post-treatment periods limits the reliability of the method. DID analyses should be supplemented by additional empirical strategies or robustness checks to enhance the credibility of the findings. Among complementary approaches, the SCM has emerged as a useful alternative, particularly for case studies. However, the SCM has notable limitations. Specifically, when the pre-treatment characteristics of the treated unit cannot be approximated by the convex combination of the characteristics of the control units, it becomes difficult to construct a credible counterfactual, thereby restricting the applicability of the SCM in some contexts.

To address problems with past methods, Arkhangelsky et al. [78] proposed the SDID method, which represents a significant advancement in both theory and practice. The SDID method not only reduces the strong need for the parallel trends assumption in the DID model but also includes fixed effects for each unit. For this reason, it can be used in more cases, even when there is more than one treated group. As a result, the SDID method is more reliable and provides better estimates, which makes it a better choice for policy evaluation under certain conditions.

A balanced panel dataset of N individuals and T periods is considered in this study. ${W_{it}}$ is a binary treatment indicator, and ${Y_{it}}$ is the outcome variable of individual i in period t. In this model, there are ${N_{co}}$ untreated individuals and ${N_{tr}}=N - {N_{co}}$ treated individuals. The constant term, individual fixed effects, and time fixed effects are denoted by the terms $\upmu, {\upalpha _i},{\upbeta _t}$, respectively. *τ * represents the treatment effects. Variables with a “hat” notation indicate the estimated values. Equation (11) indicates that the two-way fixed effects regression of the SDID estimation retains both the two-way fixed effects ${\upalpha _i}, {\upbeta _t}$ of the DID method and the individual weight $\hat {\upomega }_{i}^{{sdid}}$ of the SCM. In addition, a time weight $\hat {\uplambda }_{t}^{{sdid}}$ is introduced. Compared with these two methods, the SDID method is more general.

11 $$\begin{aligned}({\hat {\uptau }^{sdid}},\hat {\upmu },\hat {\upalpha },\hat {\upbeta })\\=\mathop {argmin}\limits_{{\uptau ,\upmu ,\upalpha ,\upbeta }} \left\{ {\sum\limits_{{i=1}}^{N} {\sum\limits_{{t=1}}^{T} {{{({Y_{it}} - \mu - {\alpha _i} - {\beta _t} - {W_{it}}\uptau )}^2}} } } \right.\left. {\hat {\upomega }_{i}^{{sdid}}\hat {\uplambda }_{t}^{{sdid}}} \right\}\end{aligned}$$

By introducing individual weights and time weights, the SDID framework enhances the construction of a credible counterfactual. In this setting, higher weights are often given to individuals who were more like the treatment group before the treatment and at times that were more like the post-treatment period. This weighting mechanism ensures that in the overall pre-treatment periods, the weighted control group closely resembles the arithmetic average of the treatment group. Furthermore, the pre-treatment weighted average of each control unit systematically differs from its post-treatment weighted average, facilitating a robust comparison framework.

The flexibility of the model is increased by adding unit fixed effects, which allow for heterogeneous baseline trends across units. This adjustment increases the robustness and reliability of the model, particularly in observational settings. By expanding the estimator to capture time-varying treatment effects, we build the SDID framework to identify a distinct treatment effect for each post-treatment period. This dynamic specification is particularly well-suited for assessing policies with delayed impacts or impacts that evolve over time. The formal expression for the dynamic SDID estimator is shown as Eq. (12):

12 $$ \begin{aligned}{\hat{\uptau }}_{{t^{'} }}  = \left[ {\frac{1}{{N_{{tr}} }}\sum\limits_{{i = N_{{co}} + 1}}^{N} {(Y_{{it}} - \sum\limits_{{t = 1}}^{{T_{{pre}} }} {{\hat{\lambda }}_{t}^{{sdid}} Y_{{it}} } } )} \right] \quad\!\!\!\!\!\!\!-\! \left[ {\sum\limits_{{i = 1}}^{{N_{{co}} }} {{\hat{\omega }}_{i}^{{sdid}} (Y_{{it}} - \sum\limits_{{t = 1}}^{{T_{{pre}} }} {{\hat{\lambda }}_{t}^{{sdid}} Y_{{it}} )} } } \right] \end{aligned}$$

where t^′^ represents one post-treatment period. The findings of each treated individual are compared to those of the resulting control individual using the SDID estimation approach. The weighted average term for the processing individual is $\frac{1}{{{N_{tr}}}}\sum\limits_{{i={N_{co}}+1}}^{N} {({Y_{it}} - \sum\limits_{{t=1}}^{{{T_{pre}}}} {\hat {\uplambda }_{t}^{{sdid}}{Y_{it}}} } )$. The weighted average of all individual differences is computed when there are ${N_{tr}}$ individuals in the sample; the weight is $\frac{1}{{{N_{tr}}}}$.

**Table 6:** Synthetic difference-in-differences regression results

VARIABLES	(1)	(2)
	DID_DATFP	SDID_DATFP
CETR	0.2425**	0.1922***
	(0.0895)	(0.0746)
[95% Conf. Interval]	[0.0671, 0.4179]	[0.0460 0.3384]
Control variables	NO	NO
Year FE	YES	YES
Province FE	YES	YES
Observations	360	360

Standard errors are in parentheses. *** *p* < 0.01, ** *p* < 0.05, * *p* < 0.1

To further confirm the robustness of the findings, we use the SDID approach and its dynamic extension to evaluate how the policy affects the eco-efficiency of digital agriculture. Table 6 displays the findings and reveals a static average treatment effect of 0.1922. This result is statistically significant at the 1% level and offers compelling evidence that the policy increased the eco-efficiency of digital agriculture at the provincial level. The reliability and robustness of the main conclusion of this study are strengthened by these findings.

#### Placebo Tests

To further assess the robustness of the empirical data and to examine the possibility of omitted variable bias, a placebo test is employed. This test entails randomly assigning the carbon emissions trading pilot policy to the provinces in the treatment group. Specifically, using post-policy period data (the year 2014), a set of pseudo-treatment groups is constructed through random allocation. The policy effect within these randomly chosen groups is then computed via the DID model. Under the assumption of no actual treatment, the estimated coefficient of CETR should not significantly differ from zero. If it does not, it would suggest potential model misspecification or omitted variable bias.

**Fig. 4:**
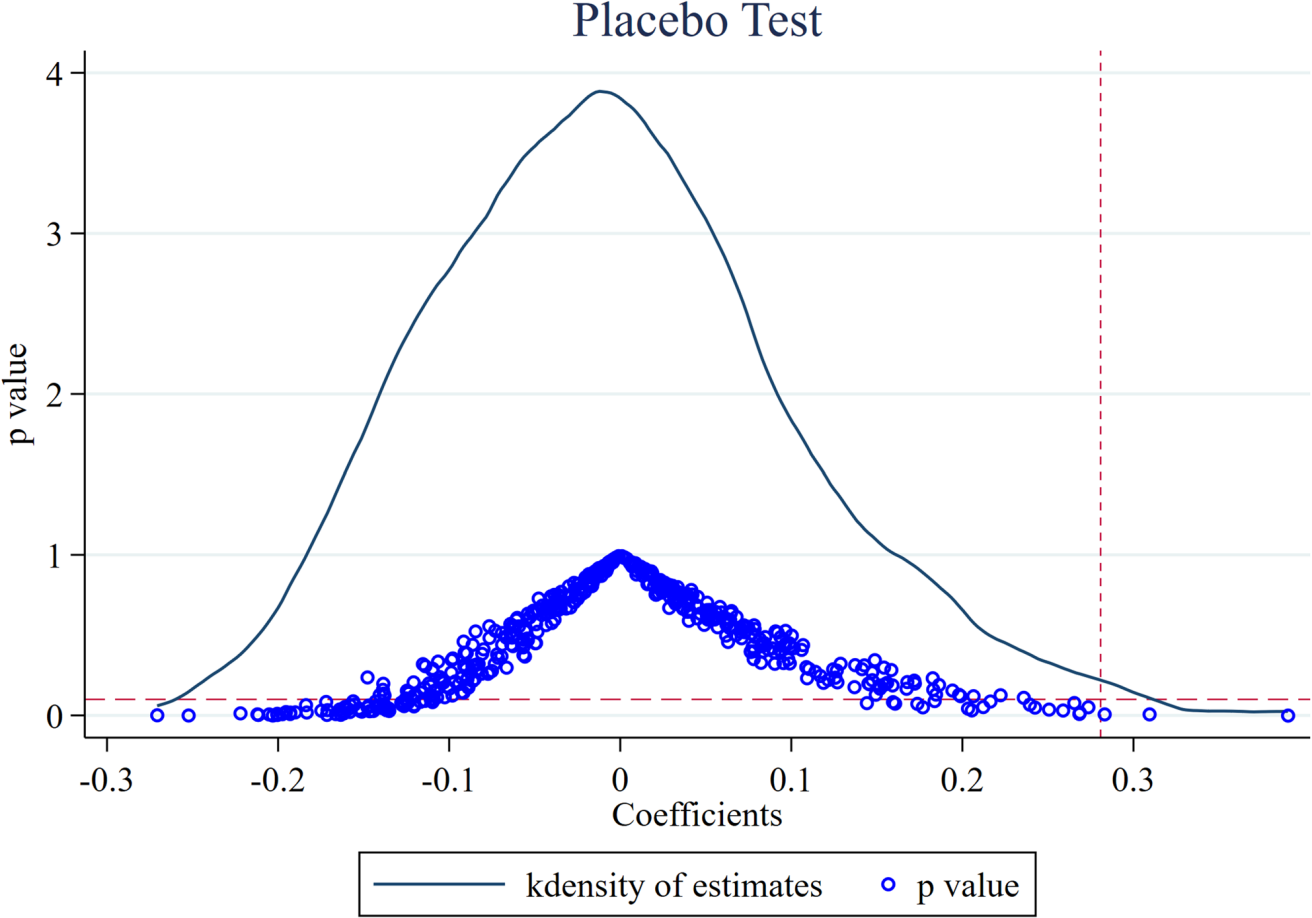
P values - estimated interaction term coefficients

The results are shown in Fig. 4. This randomization process is carried out 500 times to increase the stability of the test. As shown, the mean coefficient of the pseudo-treatment groups centres closely around zero, indicating that the randomly generated “pseudo-processing group” has no policy effect. The vertical lines in Fig. 4 represent the actual estimated coefficient, which is the outlier. The result that missing data do not substantially skew the impact of the carbon emissions trading pilot policy on the ecological efficiency of digital agriculture supports the validity of the primary empirical findings.

#### Propensity Score Matching (PSM)

In this quasi-natural experimental design, the counterfactual outcomes for the treatment group are unobservable. The assessment of the policy effect is inaccurate if there is no inherent comparability between the experimental and control groups. If there exist significant differences between these groups before the intervention, the treatment impact estimate might be biased. To address this potential issue and to mitigate endogeneity stemming from observable individual heterogeneity, this study uses the PSM method. PSM compares treated and control groups with comparable propensity scores and calculates the likelihood of treatment assignment based on observable variables, thereby constructing a more balanced sample. PSM lessens selection bias and increases the precision of the causal inference made from the DID model.

**Fig. 5:**
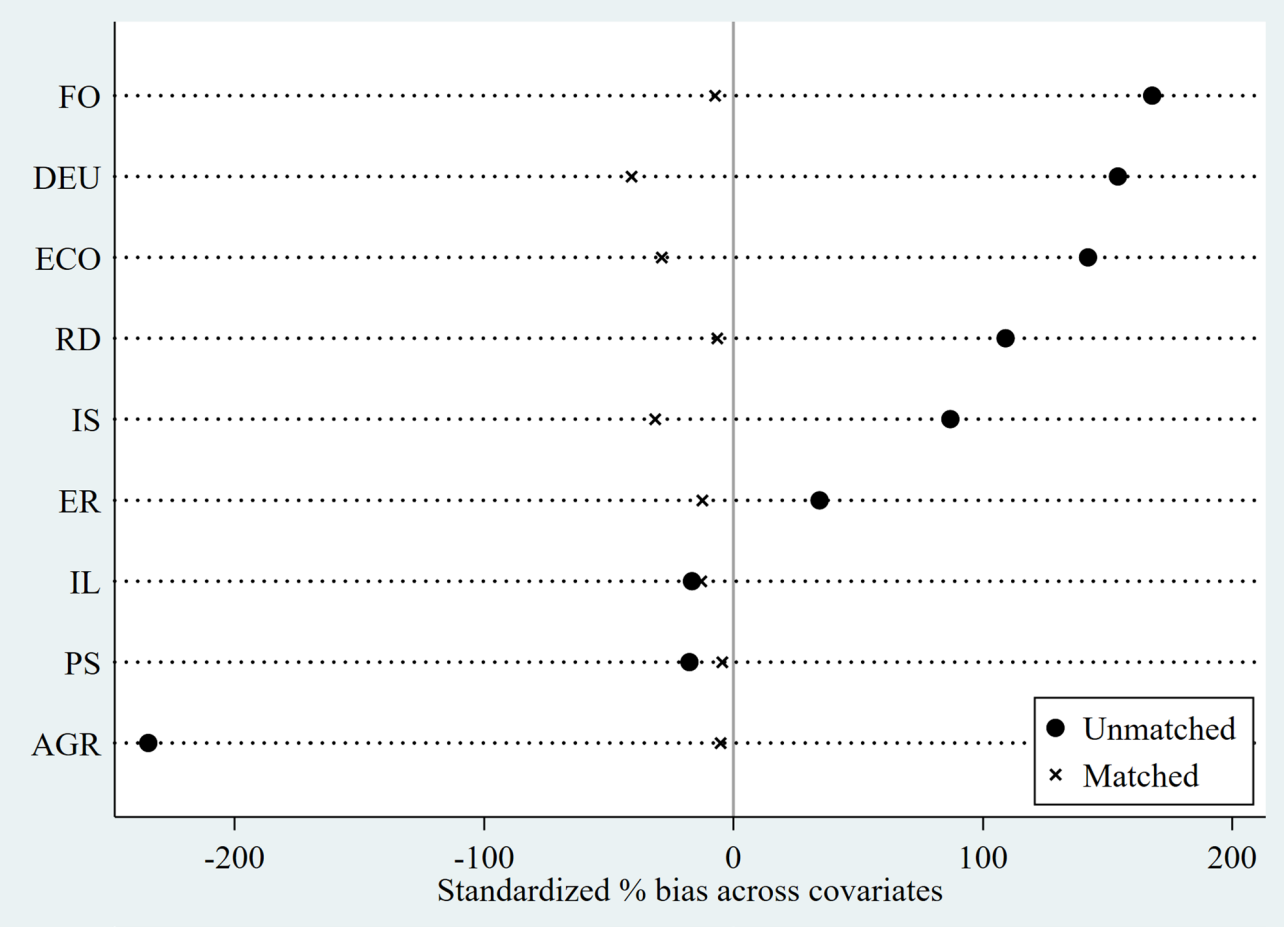
Diagram of the standardize bias of each variable

The effectiveness of the matching procedure is illustrated in Fig. 5, which presents the standardized bias of the key covariates before and after matching. Following the matching process, the data show a considerable decrease in standardized differences, suggesting that the balance between the two groups has improved. This finding reinforces the robustness of the main results and offers support for the policy effect estimation. The PSM-DID regression findings are shown in Table 7. The computed coefficients of CETR in Columns (1), (2), and (3) are statistically significant at the 1%, 5%, and 5% levels, respectively. These positive and significant results consistently strengthen the validity of the core conclusion of this study. In other words, the implementation of this policy has significantly promoted ecological efficiency within the domain of digital agricultural development.

**Table 7:** Propensity score matching coefficient estimation results

VARIABLES	(1)	(2)	(3)
	PSM_DATFP	PSM_DATFP	PSM_DATFP
CETR	0.1567***	0.1197**	0.1280**
	(0.0233)	(0.0448)	(0.0550)
Constant	1.0729***	1.0033***	0.8716
	(0.0033)	(0.0295)	(4.6238)
Control variables	NO	NO	YES
Year FE	NO	YES	YES
Province FE	YES	YES	TES
Observations	148	148	148
R-squared	0.0040	0.8920	0.8960

Standard errors are in parentheses. *** *p* < 0.01, ** *p* < 0.05, * *p* < 0.1

### Heterogeneity analysis

In this section, heterogeneity analysis is conducted from two perspectives: the geographic area and the distribution of digital agricultural eco-efficiency.

#### Regional heterogeneity

The preceding section focuses mainly on the effects of the carbon emissions trading pilot policy at the national level. However, as a developing country characterized by substantial regional economic disparities, China presents varying conditions that may lead to heterogeneous policy effects across different regions. A regional heterogeneity analysis is conducted in this study using the classification standard of the National Bureau of Statistics, which divides China into eastern, central, and western regions [79–81]. The eastern region benefits from advantageous geographic conditions, strong technological capacity, advanced management practices, and a robust economic base. The central region exhibits a moderate degree of economic growth, lying between the inland west and the coastal east. In contrast, the western region, which developed later, faces challenges because of its limited technological and economic infrastructure, but it possesses rich natural resources and substantial development potential. According to this categorization, Guangdong, Shanghai, Tianjin, and Beijing are in the eastern region, and Hubei and Chongqing are in the central and western regions, respectively.

The regression results of the policy’s varying effects in various regions are displayed in Columns (1) through (3) of Table 8. The eastern region’s computed CETR coefficient, 0.3687, is statistically significant at the 1% level, suggesting a strong positive policy effect. Significant at the 5% level, the central region’s coefficient of 0.2080 indicates a moderate but substantial influence. However, the coefficient for the western region is -0.0597 and is not statistically significant, indicating that there is limited or no policy effect in that region. Furthermore, we employed the bootstrap method to validate regional heterogeneity. The observed differences have been proven to be statistically significant at the 5% level by the results from the empirical p-value. These findings suggest that the effect of the policy on the eco-efficiency of digital agriculture varies considerably across different regions in China, thereby providing empirical support for Hypothesis 2.

**Table 8:** Results of the regional heterogeneity test

VARIABLES	(1)	(2)	(3)
	DATFP	DATFP	DATFP
CETR	0.3687***	0.2080**	-0.0597
	(0.1005)	(0.0827)	(0.1557)
Constant	-1.7393	1.0283	6.2447
	(3.9151)	(3.8913)	(11.6112)
Control variables	YES	YES	YES
Year FE	YES	YES	YES
Province FE	YES	YES	YES
Observations	144	108	107
R-squared	0.5970	0.8360	0.6450
Observations	12	9	9
Coefficient group difference test P value	-0.2901**		

Standard errors are in parentheses. *** *p* < 0.01, ** *p* < 0.05, * *p* < 0.1. To identify whether the difference in the coefficient between areas was significant, the “empirical P value” was employed and was obtained by self-sampling (bootstrapping) 500 times

#### Heterogeneity of the distribution of digital agricultural eco-efficiency

Conditional mean reversion is the fundamental idea behind general panel regression, which is based on the ordinary least squares (OLS) estimation concept. The extent to which the explained variables are impacted at various quantiles, however, may be captured by quantile regression. To further examine the heterogeneity of policy effects across the distribution of digital agricultural eco-efficiency, this study followed the approach of Kilinc-Ata and Topal [82] and adopted a quantile regression approach. Unlike traditional mean-based regressions, quantile regression allows us to estimate the policy impact at different points (quantiles) of the efficiency distribution. Doing so enables a more nuanced understanding of whether the policy has differential effects on regions with low, medium, or high levels of digital agricultural performance. The estimation results provide evidence of asymmetric policy effectiveness, revealing whether marginal returns to environmental regulation vary with the baseline eco-efficiency level.

**Table 9:** Quantile regression results

VARIABLES	(1)	(2)	(3)	(4)	(5)
	DATFP_0.1	DATFP_0.25	DATFP_0.5	DATFP_0.75	DATFP_0.9
CETR	0.0473	0.0381	0.0314	0.1790***	0.2010*
	(0.0846)	(0.0406)	(0.0389)	(0.0448)	(0.107)
Constant	-0.2250***	-0.1190***	-0.0241**	0.0870***	0.2120***
	(0.0218)	(0.0105)	(0.0101)	(0.0116)	(0.0275)
Control variables	YES	YES	YES	YES	YES
Year FE	YES	YES	YES	YES	YES
Province FE	YES	YES	YES	YES	YES
Observations	360	360	360	360	360

Notes: Standard errors are in parentheses. *** *p* < 0.01, ** *p* < 0.05, * *p* < 0.1

**Fig. 6:**
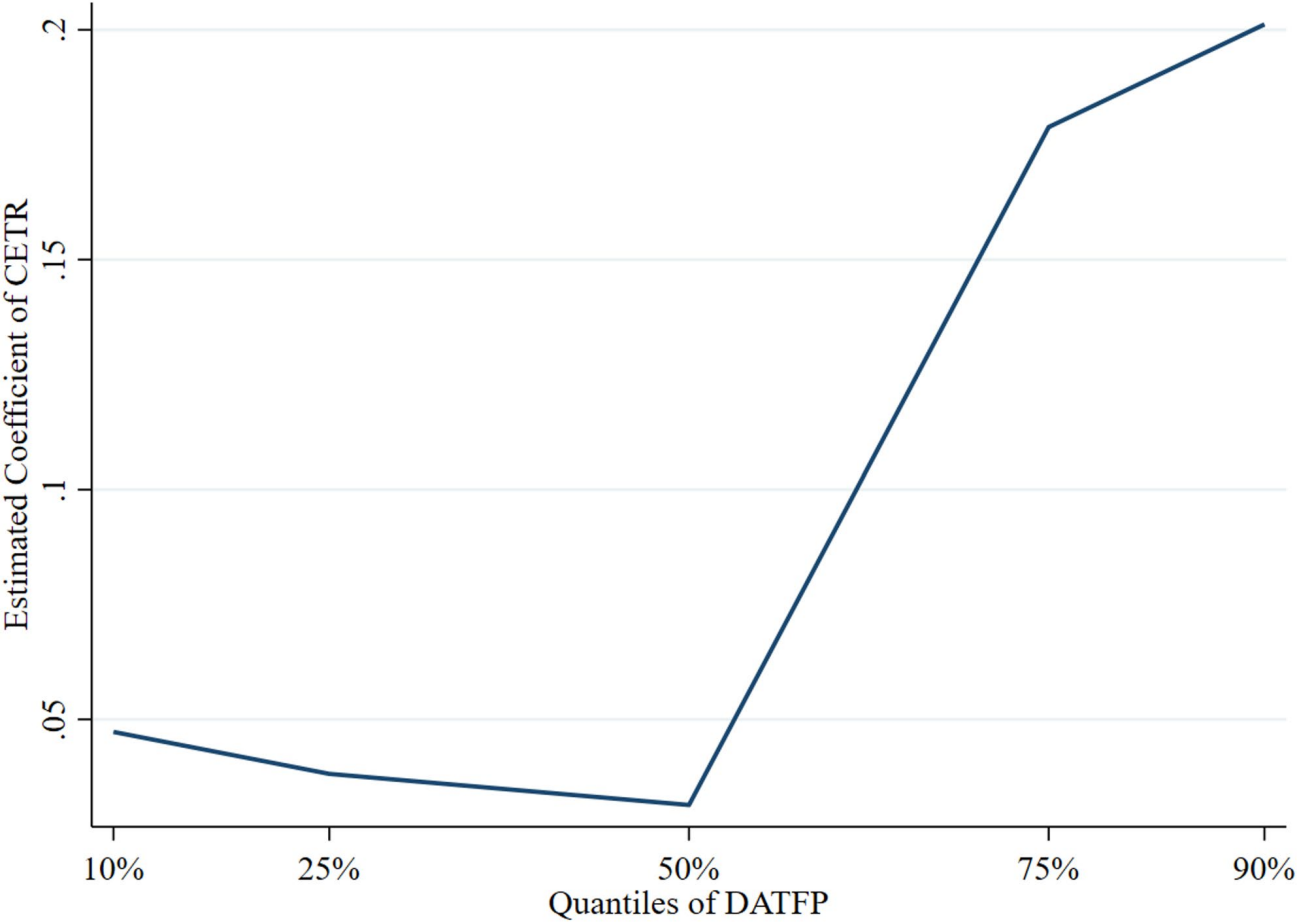
Quantile Regression: Effect of the Carbon Emissions Trading Pilot Policy

The quantile regression coefficient graph is displayed in Fig. 6, following the quantile regression findings reported in Table 9. The results indicate notable variation in the effects of the carbon emissions trading pilot policy across the distribution of digital agricultural eco-efficiency. While the estimated treatment effects are statistically non-significant at lower quantiles (10th, 25th, and 50th), they become significant and positive at the 75th and 90th percentiles. This pattern suggests that the effectiveness of the policy tends to be more pronounced in regions with higher baseline eco-efficiency levels. In such areas, relatively better technological infrastructure and stronger digital capabilities may enable a more effective response to carbon market incentives, thereby potentially amplifying the benefits of environmental regulation. Conversely, regions at the lower end of the eco-efficiency spectrum appear to be less responsive, possibly due to institutional and technological constraints, highlighting the potential need for differentiated support mechanisms to reduce regional disparities in green transformation outcomes.

### Mechanism analysis

The three-step technique states that the direct effect is c’, the mediating effect is a×b, and the total effect is c. If c’ is not significant but a and b are, then the mediating variable fully mediates the effect of the independent variable on the dependent variable. If c’ is significant and a and b are significant, the mediating variable partially mediates the impact of the independent variable on the dependent variable; if a or b is not significant, the mediating variable has no mediating effect. According to the data in Table 10, c’=0.1999, a = 0.0823, and b = 0.9827 are all significant, indicating that forest coverage plays a partial mediating role.

The carbon emissions trading pilot policy can promote the growth of forest cover as a form of natural capital through economic incentives and market mechanisms. The improvement in forest coverage, through the various ecosystem services it provides, may contribute to optimizing the natural environment on which agricultural production depends. In such an improved environment, digital agricultural technologies may be better positioned to play a role in precision management and resource optimization, thereby potentially contributing to improvements in the eco-efficiency of the agricultural system. In this process, forest coverage may play a role in transforming the “emission reduction pressure” of environmental regulation into an “ecological dividend”, thereby supporting the “green and efficient development” of agriculture.

**Table 10:** Mediating effect analysis

VARIABLES	(1)	(2)	(3)
	DATFP	FOREST	DATFP
FOREST			0.9827**
			(0.4701)
DID	0.2808**	0.0823**	0.1999*
	(0.1155)	(0.0368)	(0.1144)
Constant	-1.9140	2.1214***	-3.9987
	(3.7602)	(0.6945)	(3.9570)
Control variables	YES	YES	YES
Year FE	YES	YES	YES
Province FE	YES	YES	YES
Observations	360	360	360
R-squared	0.6310	0.6670	0.6390
ID Number	30	30	30

Standard errors are in parentheses. *** *p* < 0.01, ** *p* < 0.05, * *p* < 0.1

## Discussion

Evidence from this study suggests that the carbon emissions trading pilot policy contributes to improvements in the eco-efficiency of digital agriculture, in line with studies showing that market-based environmental regulation can simultaneously reduce emissions and stimulate green innovation [83–85]. For example, Peng et al. [86] reveal that compared with non-pilot cities, China’s ETS promotes green innovation cooperation through the upgrading effect of industrial structure and the coverage effect of digital finance at the city level, while Yu et al. [87] and Cheng and Wang [88] report that this policy promotes green innovation among firms. Related research on carbon pricing and technological upgrading further supports the Porter hypothesis, suggesting that stricter regulation can induce innovation and productivity gains. Extending this logic to agriculture implies that the ETS may not only reduce emissions but also contribute to enhancing eco-efficiency within digitally enabled production systems.

This interpretation is also broadly consistent with work emphasizing the sustainability gains from digitalization. Xu et al. [89] argue that digital strategy improves environmental performance. In this context, carbon pricing appears to reinforce the environmental dividends of digital transformation in agriculture, strengthening the synergy between digital inputs and low-carbon outcomes. Distinct from studies centred on aggregate agricultural emissions or industrial carbon intensity, the analysis identifies a potential pathway linking the ETS policy to digital agricultural eco-efficiency. Prior research has mainly evaluated emission reduction, sectoral heterogeneity, or macro-level green transition without explicitly isolating digital agriculture as a production paradigm [90, 91]. By constructing a dynamic DEA-Malmquist index, the results suggest that the ETS policy may shape not only emission outcomes but also multi-dimensional efficiency performance that integrates digital inputs with environmental outputs.

Regional heterogeneity further complements the literature. Earlier studies note that carbon markets tend to perform better in regions with stronger institutional capacity and technological endowments [92]. Consistent with this view, relatively stronger effects are observed in economically advanced eastern provinces, suggesting that digital infrastructure and market maturity may condition policy effectiveness and highlighting the importance of local absorptive capacity. The mechanism analysis also broadens the current understanding by identifying forest coverage as a potential mediating channel. While many studies link carbon trading to green innovation or renewable energy adoption [93], ecosystem-based pathways in agriculture remain underexplored. Overall, this study integrates the literature on carbon trading, digital agriculture, and eco-efficiency, suggesting that digital agriculture may exhibit higher environmental efficiency under the carbon emissions trading pilot policy, with effects that vary across regions.

## Conclusion and policy implications

### Summary of the major findings

Given the difficulties posed by climate change, it is critical to recognize that agriculture substantially contributes to greenhouse gas emissions. Against this backdrop, an index system was constructed to measure the eco-efficiency of digital agriculture, and a dynamic DEA-Malmquist index combined with a DID framework was applied to 30 Chinese provinces from 2011 to 2022. The evidence indicates that the carbon emissions trading pilot policy significantly enhances digital agricultural eco-efficiency, with robust results across specifications. The policy impacts vary across regions and development stages and operate partly through ecological channels such as increased forest coverage. More importantly, the findings suggest that carbon emissions trading functions not only as an emission-control instrument but also as an institutional catalyst linking digital transformation, ecosystem restoration, and productivity upgrading in agriculture. This implies that achieving agricultural carbon neutrality requires coordinated policies that integrate carbon markets, digital infrastructure, and ecological governance rather than relying on single-policy interventions. Although nationwide expansion faces implementation challenges, such an integrated framework provides a more viable pathway for low-carbon agricultural transformation in China. Future research should further explore micro-level adoption mechanisms and long-term market stability to refine this integrated policy framework.

### Policy implications for the agricultural green transition

To enhance flexibility and adaptability, the first policy recommendation is to enhance the function and design of the ETS mechanism. In line with Hypothesis 1, which shows that the carbon emissions trading pilot policy significantly improves the eco-efficiency of digital agriculture, strengthening the ETS market mechanism is essential to sustain and amplify this positive effect [94]. Promoting a green transformation in agriculture by offering market-based incentives is among the most important objectives of the ETS. However, as the economy changes and technology develops faster, the carbon emissions trading market faces new challenges and higher demands. These include fluctuations in carbon prices, different levels of technological development in each region, and the need for better ways to measure carbon. Therefore, it is important to improve how quotas are distributed, induce more people to become involved in the market, and make the rules clearer and easier to follow. Performing these tasks will help keep the system strong and ensure that it is able to support low-carbon growth in agriculture. Making the market work better and making rules more flexible will help fight climate change, cut emissions, and support better economic growth. In addition, to make the quota allocation fairer and more useful, a standard method should be used. Using such a method will push companies to lower their emissions. The rules for allocating quotas should change based on how much each company produces and how much it can reduce. A shared platform should also be made to provide clear and equal information, which will help make the market more open, reduce confusion, and lead to more trading and more trust among market players [95, 96].

Another suggestion is to better match policies to support the use of digital agriculture and green technologies [97]. Because Hypothesis 1 confirms that the ETS promotes eco-efficiency partly through technological upgrading and ecological pathways, complementary policies are needed to reinforce these transmission channels. The carbon emissions trading pilot policy should not operate in isolation; instead, it should be coordinated with other policy tools to facilitate the comprehensive green transformation of digital agriculture. Specifically, the carbon emissions trading pilot policy should be integrated with agricultural subsidy policies and ecological compensation mechanisms. Agricultural subsidies focus primarily on production and provide limited incentives for the adoption of green technologies. The following measures are recommended to address this gap. Green subsidies offer farmers and agricultural businesses direct incentives to adopt environmentally friendly production technology and are crucial for promoting low-carbon agricultural growth. Recent evidence also shows that integrating land consolidation with smart agriculture technologies can significantly enhance agricultural carbon neutrality pathways and food security, highlighting the importance of coordinated policy design between carbon markets and digital agriculture development [98].

Finally, enhancing region-specific policy strategies and optimizing the carbon emissions trading framework are important [99]. Consistent with Hypothesis 2, which identifies significant regional heterogeneity in policy effectiveness, differentiated regional strategies are necessary to achieve equitable and efficient outcomes. Since the policy works differently in each region, different regions need their own plans to obtain more effective and equitable outcomes. The eastern region has strong economic foundations and high levels of technological innovation; it experiences the most pronounced policy effects. Efforts there should focus on further enhancing market activity, reducing transaction costs, and improving the carbon emissions trading market mechanism. To promote smarter and more environmentally friendly agricultural production, encouraging companies to increase their investments in the research and development of green technologies is essential, as it promotes the integration of digital agriculture with low-carbon technologies. However, the western region demonstrates weaker policy impacts, which can be attributed to constraints such as limited resource endowments, less developed industrial structures, and insufficient policy enforcement capacity. To assist businesses and local governments in comprehending and implementing the carbon emissions trading pilot policy, communication and training initiatives should be reinforced. In addition, western irrigation regions can achieve this goal through efficient large-scale water-saving irrigation [100].

### Limitations and future research directions

To assess digital agricultural eco-efficiency, in this study, a dynamic DEA-Malmquist index is created, an ecological development index system for digital agriculture is constructed, and the impact of the carbon emissions trading pilot policy on the eco-efficiency of digital agriculture is analysed via the DID method. The results show that the policy has a positive effect on increasing the eco-efficiency of digital agriculture. However, research limitations remain that future studies could address. One research opportunity is to expand the dataset. This study uses available data at the provincial level, which limits the ability to capture micro-level dynamics. Future research should obtain city- or county-level data for a more comprehensive analysis. In addition, although regional heterogeneity is partially addressed, further exploration of spatial interactions remains limited. Future research could incorporate spatial econometric models to explore whether the policy’s benefits ‘leak’ into neighbouring non-pilot areas, thereby providing a more holistic view of regional collaborative emission reduction.

## Data Availability

The data and code supporting the findings of this study are openly available in Figshare at https://doi.org/10.6084/m9.figshare.31566460. The repository includes the full panel dataset (2011–2022), Stata data files, and replication code.
